# Distribution and UV protection strategies of zooplankton in clear and glacier-fed alpine lakes

**DOI:** 10.1038/s41598-017-04836-w

**Published:** 2017-07-03

**Authors:** Barbara Tartarotti, Florian Trattner, Daniel Remias, Nadine Saul, Christian E. W. Steinberg, Ruben Sommaruga

**Affiliations:** 1 Lake and Glacier Research Group, Institute of Ecology, University of Innsbruck, 6020 Innsbruck, Austria; 2 Institute of Botany, University of Innsbruck, 6020 Innsbruck, Austria; 3 Laboratory of Freshwater and Stress Ecology, Department of Biology, Humboldt-Universität zu Berlin, 12437 Berlin, Germany; 4 University of Applied Sciences Upper Austria, 4600 Wels, Austria; 5 Institute of Biology, Humboldt-Universität zu Berlin, 12437 Berlin, Germany

## Abstract

Zooplankton, a group of aquatic animals important as trophic link in the food web, are exposed to high levels of UV radiation (UVR) in clear alpine lakes, while in turbid glacier-fed lakes they are more protected. To study the interplay between behavioral and physiological protection responses in zooplankton from those lakes, we sampled six lakes of different UVR transparency and glacial turbidity. Copepods were absent in the upper water layers of the clearest lake, while in glacier-fed lakes they were more evenly distributed in the water column. Across all lakes, the weighted copepod mean depth was strongly related to food resources (chlorophyll *a* and rotifers), whereas in the fishless lakes, glacial turbidity largely explained the vertical daytime distribution of these organisms. Up to ~11-times (mean 3.5) higher concentrations of photo-protective compounds (mycosporine-like amino acids, MAAs) were found in the copepods from the clear than from the glacier-fed lakes. In contrast to carotenoid concentrations and antioxidant capacities, MAA levels were strongly related to the lake transparency. Copepods from alpine lakes rely on a combination of behavioral and physiological strategies adapted to the change in environmental conditions taking place when lakes shift from glacially turbid to clear conditions, as glacier retreat proceeds.

## Introduction

The retreat of glaciers, a consequence of recent climate change^1^, causes changes in lake water turbidity and prolonged periods of high mineral particle (i.e., glacial flour) loads during the ice-free season in small alpine lakes, while these lakes will eventually turn clear when the hydrological connectivity to the glacier is lost^2, 3^. Although zooplankton living in glacier-fed lakes are potentially protected from high levels of solar UV radiation (UVR, ~290–400 nm), they are exposed to UVR throughout the water column in clear alpine lakes, as these lakes are highly transparent to UVR^4^. In addition, many of these systems are relatively shallow (<15 m depth), thus, the extent of potential UVR avoidance is limited and will change with changes in water turbidity.

Zooplankton have evolved various strategies to minimize the harmful direct and indirect effects of solar UVR (see ref. 5 for a review). They avoid high levels of UVR by vertical downward migration during daytime^6, 7^, which is most pronounced in *Daphnia* ^8^, but can also be observed in other zooplankton taxa such as copepods^9, 10^. In a recent study, both avoidance and attraction were found in calanoid copepods in response to solar UVR^11^. Light is generally regarded as the primary proximate cue used by zooplankton to induce diel vertical migration (DVM)^12^. More recently, UVR has been included as a driver of DVM in clear lakes, even when stocked with fish^10, 13, 14^. On the other hand, it can be difficult to separate the roles of fish predation and UVR as drivers influencing the distribution of zooplankton, especially in clear lakes^14^.

Copepods accumulate photoprotective compounds such as carotenoids^15^ and mycosporine-like amino acids (MAAs)^16^ for protection against UVR. Free astaxanthin and esterified derivatives are the main carotenoids stored in copepods^15, 17^, giving them a red pigmentation. Many of these lipid-soluble pigments have strong antioxidant properties and reduce photooxidative stress induced by short-wavelength solar radiation^18^. Previous work showed that unpigmented copepods experience higher UV vulnerability than pigmented ones^19, 20^, and that the levels of carotenoids in these animals increase with elevation and decrease with lake depth^19^. Recently, very high carotenoid concentrations (free astaxanthin) were observed in copepods from clear fishless Himalayan alpine lakes^17^. The concentrations of carotenoids in these calanoid copepods were inversely related to the lake depth refuge (i.e., the fraction of the water column to which 1% of the surface irradiance at 320 nm penetrates), with the lowest concentrations found in copepods from a turbid glacier-fed lake^17^.

In the presence of visually-oriented predatory fish, carotenoids seem to be detrimental for zooplankton due to their higher visibility given by the red coloration^21^. However, several freshwater organisms, including copepods, also accumulate UV-absorbing MAAs, to effectively protect from UVR^16^. MAAs are a group of intracellular, water-soluble, low molecular weight compounds with absorption maxima in the UV wavelength region (309–360 nm). These compounds directly absorb and screen out UVR in many aquatic invertebrates, and dissipate the absorbed energy into heat without generating oxidative stress induced by reactive oxygen species (ROS)^22^. The variability in MAA concentrations of different copepod populations (mostly *Cyclops abyssorum tatricus*) from lakes located across an elevational gradient in the Central Eastern Alps is strongly related to lake elevation, UVR transparency and depth refuge^23^, supporting the idea that these compounds are essential for their survival in clear alpine lakes.

In addition to carotenoids, various antioxidants are necessary for the neutralization and quenching of UV-induced toxic photoproducts including ROS in aquatic organisms^24^. Differences in the expression of these enzymes are found between copepods and cladocerans^25^, and a rapid enzymatic response is observed in the calanoid copepod *Eudiaptomus gracilis* after exposure to UVR^26^. Most studies reporting antioxidant activities in zooplankton focused on particular enzymes^25–29^. Here, we determined the overall antioxidant capacity, providing an integrated parameter of the *in vivo* balance between ROS and antioxidant compounds^30^. The antioxidant capacity is divided into two fractions, the hydrophilic and the lipophilic one^31^. The hydrophilic antioxidant capacity protects the water soluble (e.g. proteins) and the lipophilic antioxidant capacity the lipophilic structures (e.g. membranes) of a cell. Usually, the hydrophilic fraction exceeds the lipophilic one by roughly one order of magnitude^32^.

Although several studies have addressed UV protection strategies in zooplankton from high mountain lakes^14–17, 23, 33, 34^, to our knowledge, the present study is the first to compare the combination of behavioral and physiological responses to UVR in alpine lakes across a gradient of UVR transparency ranging from glacially turbid to clear. Here, we focus on the effects of ambient levels of solar UVR on the widespread copepod *Cyclops abyssorum tatricus* (Kozminski). Our aim was to understand the interplay between the daytime vertical distribution and different protection strategies (MAAs, carotenoids, antioxidant capacities) of these organisms in relation to the lake optical properties.

## Results

### Copepod abundance and daytime vertical distribution

**Figure 1:**
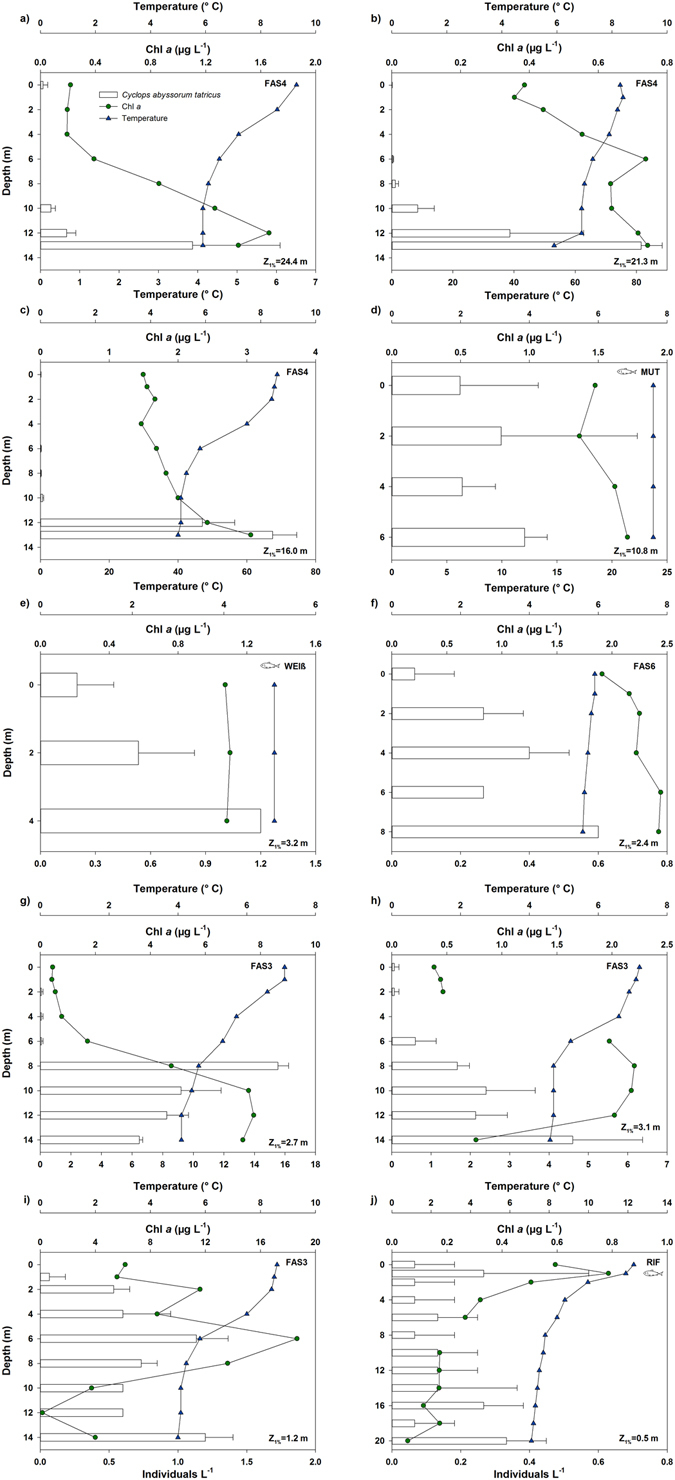
Vertical daytime distribution of *Cyclops abyssorum tatricus*, Chlorophyll *a* (Chl *a*), and temperature in six alpine lakes during the ice-free period of 2010 and 2011. The lakes are ordered from clear (i.e. no glacier connectivity, NTU < 1) to turbid (i.e. glacial influence, NTU > 1). Error bars indicate +1 standard deviation. Bars without error bars indicate a standard deviation of zero. (**a**) FAS4 July 2010, (**b**) FAS4 July 2011, (**c**) FAS4 August 2011, (**d**) MUT, (**e**) WEIß, (**f** ) FAS6, (**g**) FAS3 July 2010, (**h**) FAS3 July 2011, (**i**) FAS3 August 2011, (**j**) RIF (abbreviations for the lakes are defined in Table 1). Z_1%_ = 1% of the surface irradiance at 320 nm.

Mean abundances of *C*. *abyssorum tatricus* were ~7 times higher in the clear (9.5 Ind L^−1^) than in the glacier-fed (1.3 Ind L^−1^) lakes. In the clearest lake (Faselfadsee 4, FAS4), the copepods were mainly distributed close to the bottom of the lake (Fig. 1a–c), while in the most turbid lakes, the copepod distribution showed no clear maximum (Fig. 1i,j). In the highly turbid Lake Rifflsee (RIF), copepods were nearly equally distributed and had very low abundances throughout the water column (Fig. 1j). Similarly, at the time of highest water turbidity in Lake Faselfadsee 3 (FAS3) (August 2011; Table 1), copepods were almost evenly distributed in the water column, except for the water surface (Fig. 1i). In the other glacier-fed lakes, the *C*. *abyssorum tatricus* populations showed a preference for the middle to deeper water depths (Fig. 1e–h). In fish-stocked, clear Lake Mutterbergersee (MUT), copepods were found in all water layers with similar abundance (Fig. 1d).

**Table 1:** Main characteristics of the study lakes including the day of sampling, altitude, lake area, maximum lake depth (z_max_), water optical properties (mean dissolved organic carbon content (DOC), mean turbidity (nephelometric turbidity units, NTU; min/max), depth of 1% of surface irradiance for 320 nm UV (Z_1%320_), and fraction of the water column to which 1% of the surface irradiance at 320 nm penetrated [Z_1%320_/Z_max_]) on the day of sampling, mean pH, and fish species present in the lakes.

Lake/Sampling date	Altitude (m.a.s.l.)	Area (ha)	Z_max_ (m)	Direct glacier influence	DOC (µg L^−1^)	Mean Turbidity (NTU)	Z_1%320_ (m)	Z_1%320_/Z_max_	pH	Fish species
Faselfadsee 4 (FAS4)	2416	1.9	15.0	No						
2010-07-12					245	0.24 (0.13/0.34)	24.4^+^	1.63	7.6	
2011-07-05					299	0.22 (0.17/0.26)	21.3^*^	1.42	7.4	
2011-08-29					305	0.17 (0.13/0.22)	16.0^*^	1.07	7.3	
Mutterbergersee (MUT)	2483	3.8	7.5	No						*S*. *alpinus*
2010-09-23					523	0.67 (0.54/0.83)	10.8^+^	1.44	6.2	
Weißsee (WEIß)	2425	1.5	6.3	Yes						*S*. *trutta* f. *fario*, *S*. *alpinus*
2010-10-10					313	1.36 (1.30/1.40)	3.2^+^	0.51	7.6	
Faselfadsee 6 (FAS6)	2263	1.3	10.0	Yes						
2010-09-14					176	4.73 (4.58/4.89)	2.4^+^	0.24	7.6	
Faselfadsee 3 (FAS3)	2420	2.1	17.0	Yes						
2010-07-12					227	3.19 (0.96/4.50)	2.7^+^	0.16	7.5	
2011-07-05					165	4.38 (3.91/4.63)	3.1^*^	0.18	7.6	
2011-08-29					267	8.57 (5.82/11.37)	1.2^*^	0.07	8.0	
Rifflsee (RIF)	2234	26.9	24.0	Yes						*S*. *trutta* f. *fario*, *O*. *mykiss*, *S*. *alpinus*
2010-08-23					284	48.9 (38.40/58.55)	0.5^+^	0.02	7.1	

(*S*. *alpinus*: *Salvelinus alpinus*; *S*. *trutta* f. *fario*: *Salmo trutta* forma *fario*; *O*. *mykiss*: *Oncorhynchus mykiss*). Z_1%320_ derived from PUV-501B depth profiles (^*^) or CDOM (^+^), respectively.

**Figure 2:**
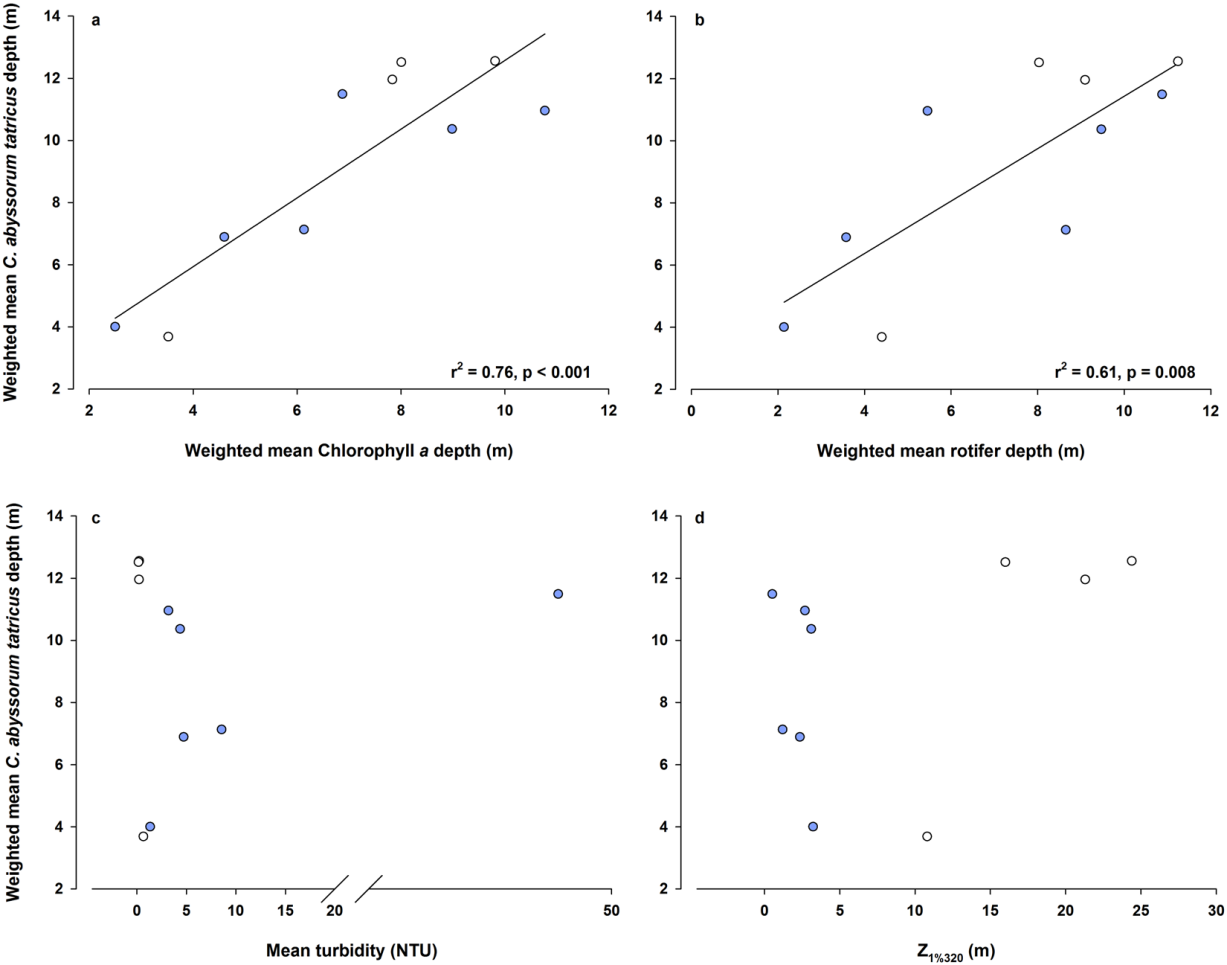
Weighted mean *Cyclops abyssorum tatricus* depth vs. (**a**) the weighted mean Chlorophyll *a* (Chl *a*) depth, (**b**) weighted mean rotifer depth, (**c**) mean turbidity, and (**d**) 1% of the surface irradiance at 320 nm (Z_1%320_). Open circles: clear lakes, closed circles: turbid lakes.

**Figure 3:**
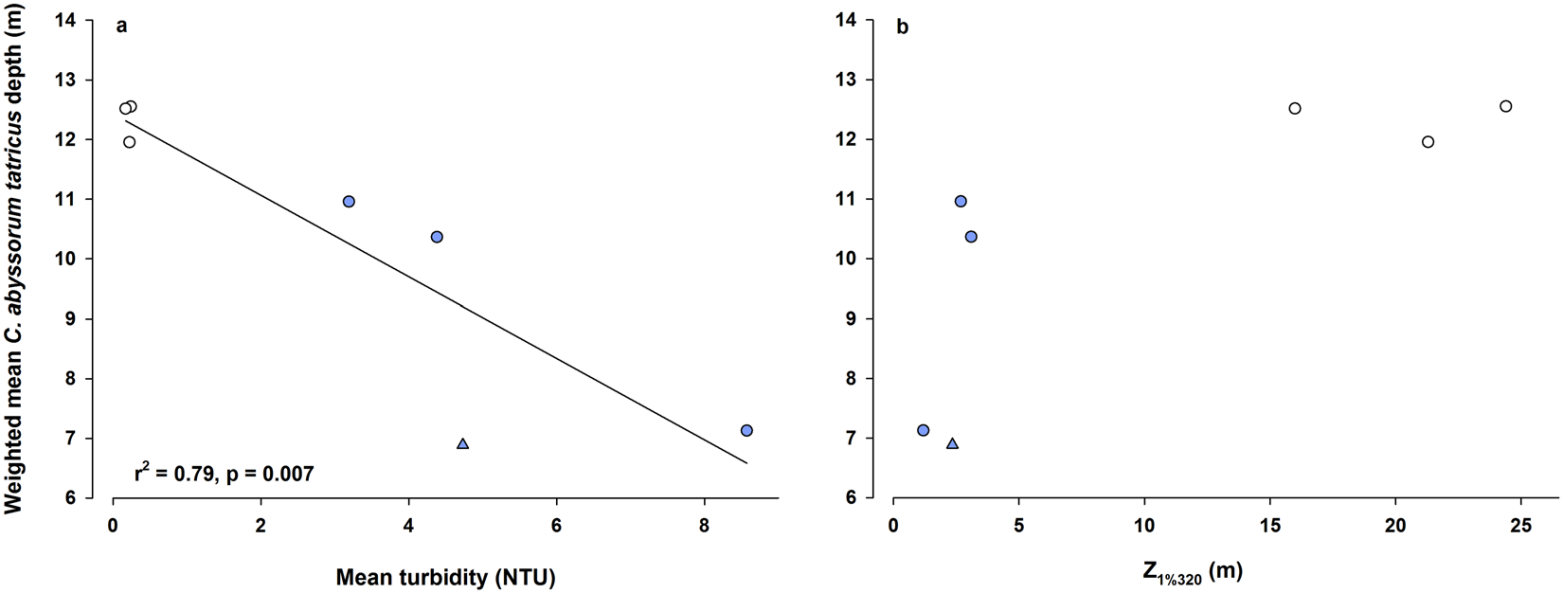
Weighted mean *Cyclops abyssorum tatricus* depth vs. (**a**) the mean turbidity and (**b**) 1% of the surface irradiance at 320 nm (Z_1%320_) when considering only the three study lakes without fish. Open circles: clear lake FAS4, closed circles: turbid FAS3, and closed triangles: turbid FAS6 (abbreviations for the lakes are defined in Table 1).

Across all study lakes, weighted mean depths of *C*. *abyssorum tatricus* were positively correlated with the weighted mean Chlorophyll *a* (Chl *a*) (r^2^ = 0.76, n = 10, p < 0.001) and weighted mean rotifer (r^2^ = 0.61, n = 10, p = 0.008) depths (Fig. 2a,b), suggesting that the distribution of the copepods was linked to their food resources. Depending on the study site, temperature at the mean depths of the copepods varied from 4.4 to 9.1 °C, showing no relationship with the weighted mean *C*. *abyssorum tatricus* depth (r^2^ = 0.05, n = 10, p = 0.547). Similarly, parameters such as turbidity or the depth of 1% of the surface irradiance at 320 nm (Z_1%320_) showed no relationship (Fig. 2c,d). While Z_1%320_ and weighted mean Chl *a* or mean dissolved organic carbon (DOC) concentrations were not significantly correlated across lakes (Chl *a*: r^2^ = 0.10, n = 10, p = 0.383; DOC: r^2^ = 0.06, n = 10, p = 0.511), there was a strong negative relationship between Z_1%320_ and turbidity (r^2^ = 0.81, n = 10, p < 0.001). When considering only the lakes without fish, the *C*. *abyssorum tatricus* weighted mean depth was negatively correlated with the water turbidity (r^2^ = 0.79, n = 7, p = 0.007) (Fig. 3a), indicating that the copepods stayed deeper in the water column as turbidity decreased. The depth of 1% of the surface irradiance at 320 nm (Z_1%320_) did not show a clear trend with the copepod weighted mean depth (Fig. 3b). The mean copepod depth was again positively correlated with the mean Chl *a* depth (r^2^ = 0.57, n = 7, p = 0.049), but to a lesser and not statistically significant extent with the mean rotifer depth (r^2^ = 0.30, n = 7, p = 0.199). Again, Z_1%320_ and the mean Chl *a* depth or mean DOC concentrations were not significantly correlated (Chl *a*: r^2^ = 0.10, n = 7, p = 0.50; DOC: r^2^ = 0.36, n = 7, p = 0.156), while the Z_1%320_ was negatively correlated with the turbidity of the lakes (r^2^ = 0.96, n = 7, p < 0.001). The mean depth of *C*. *abyssorum tatricus* in the fishless lakes was deeper (10.3 m) than in the lakes with fish (6.4 m), but the difference was not statistically significant (t-test, p = 0.097).

### MAAs

**Figure 4:**
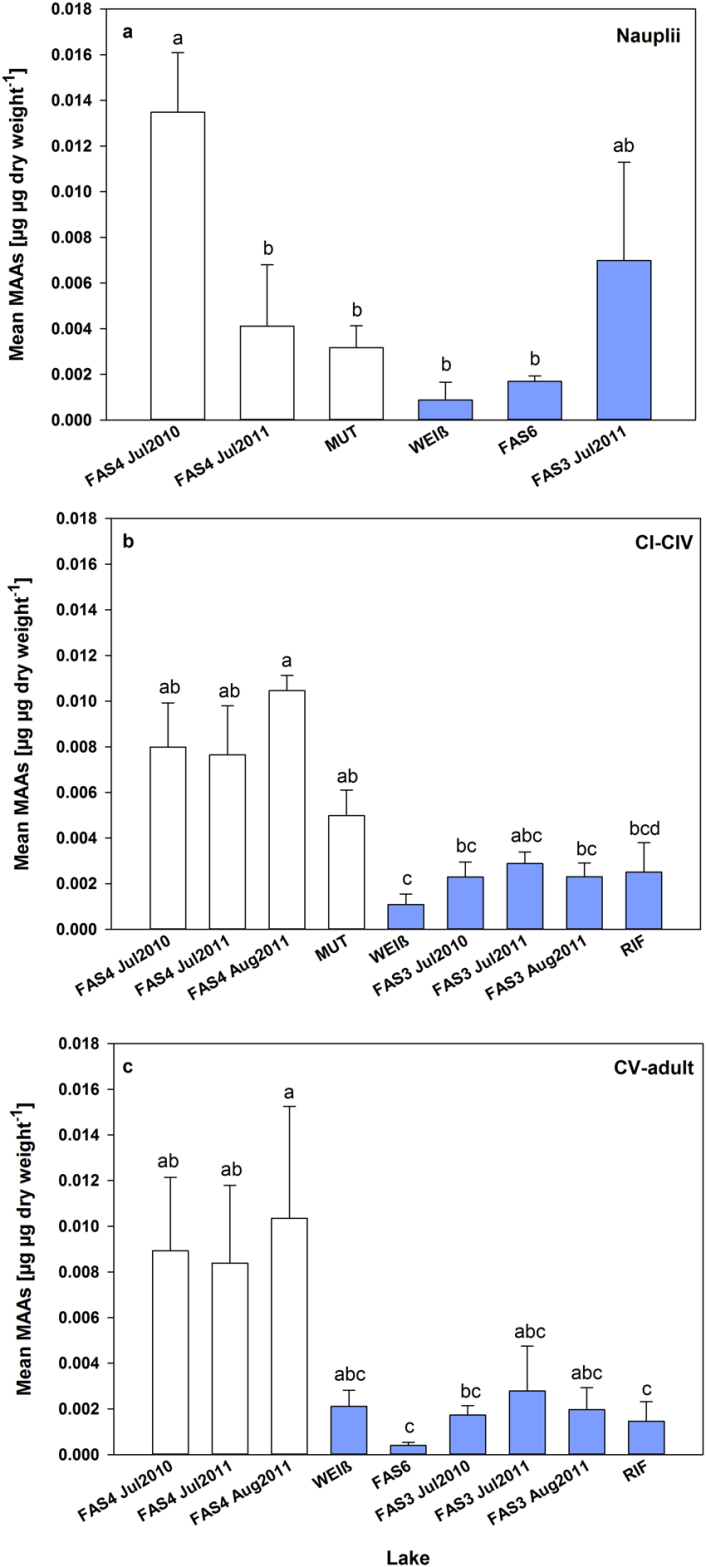
Total mean MAA concentrations (µg µg dry weight^−1^) in the different life stages nauplii, copepodid CI-CIV, and copepodid CV to adult of *Cyclops abyssorum tatricus* from six alpine lakes sampled during the ice-free period of 2010 and 2011. The lakes are ordered from clear to turbid (abbreviations for the lakes are defined in Table 1). Error bars indicate +1 standard deviation. Different letters above the bars indicate a significant difference found with one-way ANOVA all pairwise comparison procedures (Holm-Sidak method, F_5,12_ = 11.36, p < 0.001 for nauplii), and Kruskal-Wallis one-way ANOVA on Ranks, all pairwise multiple comparison procedures (Dunn’s method, p < 0.05 for CI-CIV and CV-adult). White bars: clear lakes, blue bars: turbid lakes. Nauplii were not present in all lakes at the time of sampling. Copepodid CI-CIV and CV-adult were not present in Lakes FAS6 and MUT, respectively, at the time of sampling.

**Figure 5:**
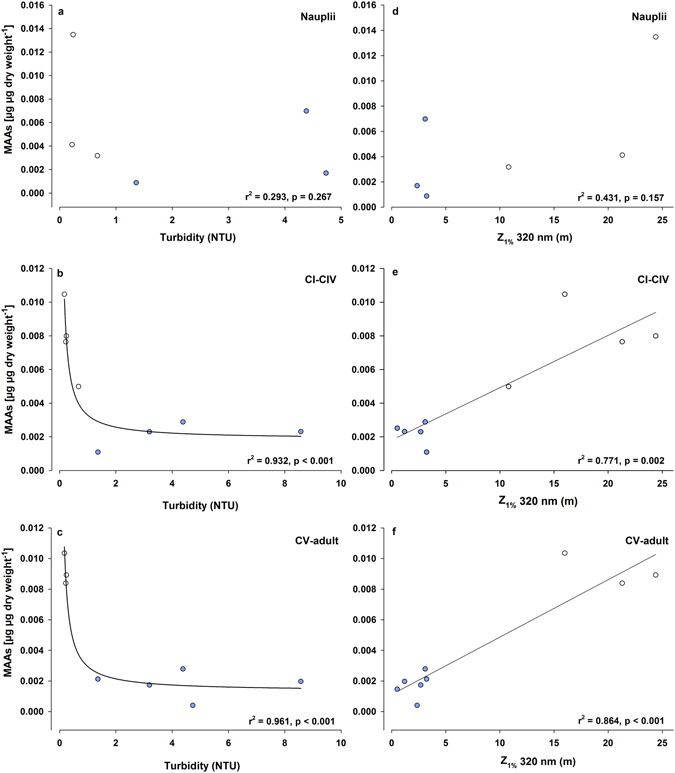
Relationship between total mean concentrations of MAAs (µg µg dry weight^−1^) in *Cyclops abyssorum tatricus* nauplii, copepodid stages CI-CIV, and copepodid stages CV to adult and the turbidity (**a**–**c**) and the 1% of the surface irradiance at 320 nm (Z_1%320_) (**d**–**f**), respectively. Turbidity data are presented without Lake RIF. Open circles: clear lakes, closed circles: turbid lakes.

Copepod samples from Lakes FAS3 and RIF, although being turbid glacier-fed lakes, contained detectable amounts of all six MAAs, while in turbid Lakes Faselfadsee 6 (FAS6) and Weißsee (WEIß) only two and three different MAAs, respectively, were detected (Table S1). Shinorine was the predominant MAA, contributing up to ~89% of the total MAA concentration (Table S1). For easier comparison, MAA concentrations from copepodid stages CI-CIV and CV-adult, respectively, were pooled (see data treatment). Mean MAA concentrations of the nauplii (July 2010), copepodid stages CI-CIV, and CV-adult life stages were significantly higher in Lake FAS4 when compared with the other study lakes (Fig. 4), except for FAS3 in July 2011. Overall, mean MAA concentrations (nauplii to adult life stages) were statistically significantly higher (~3.5-times; Mann-Whitney Rank Sum Test, p < 0.001) in the populations from the clear lakes (FAS4 and MUT) than from the glacier-fed ones. A positive relationship was found between mean MAA concentrations and the depth of 1% of the surface irradiance at 320 nm (Z_1%320 nm_) for copepodid stages CI-CIV (r^2^ = 0.77, n = 9, p = 0.002) and copepodid stage CV to adult life stages (r^2^ = 0.86, n = 9, p < 0.001), while a weaker, not significantly different relationship was observed for nauplii (r^2^ = 0.43, n = 6, p = 0.157) (Fig. 5d–f). Turbidity explained a similarly high or even higher percentage of the variability in MAA concentrations of the copepodid and adult life stages (r^2^ = 0.93, n = 8, p < 0.001 for CI-CIV, and r^2^ = 0.96, n = 8, p < 0.001 for CV to adult), whereas it was again not significantly different for nauplii (r^2^ = 0.29, n = 6, p = 0.27) (Fig. 5a–c). The concentration of the MAAs correlated with the depth refuge (*Z*_1%320_/*Z*_max_) in the young copepodid stages (CI-CIV: r^2^ = 0.60, n = 9, p = 0.014) and copepodid CV to adult life stages (r^2^ = 0.82, n = 9, p < 0.001), but there was no significant relationship with the larval life stages (nauplii: r^2^ = 0.21, n = 6, p = 0.36). As the accumulation of MAAs depends not only on the radiation exposure, but also on temperature, the MAA concentration of the copepods was related to the mean water temperature, resulting in a significant relationship for copepodid stages CI-CIV (r^2^ = 0.49, n = 9, p = 0.037), but a weaker and non-significant relationship for copepodid CV to adult life stages (r^2^ = 0.39, n = 9, p = 0.075) and nauplii (r^2^ = 0.10, n = 6, p = 0.541).

### Carotenoids

**Figure 6:**
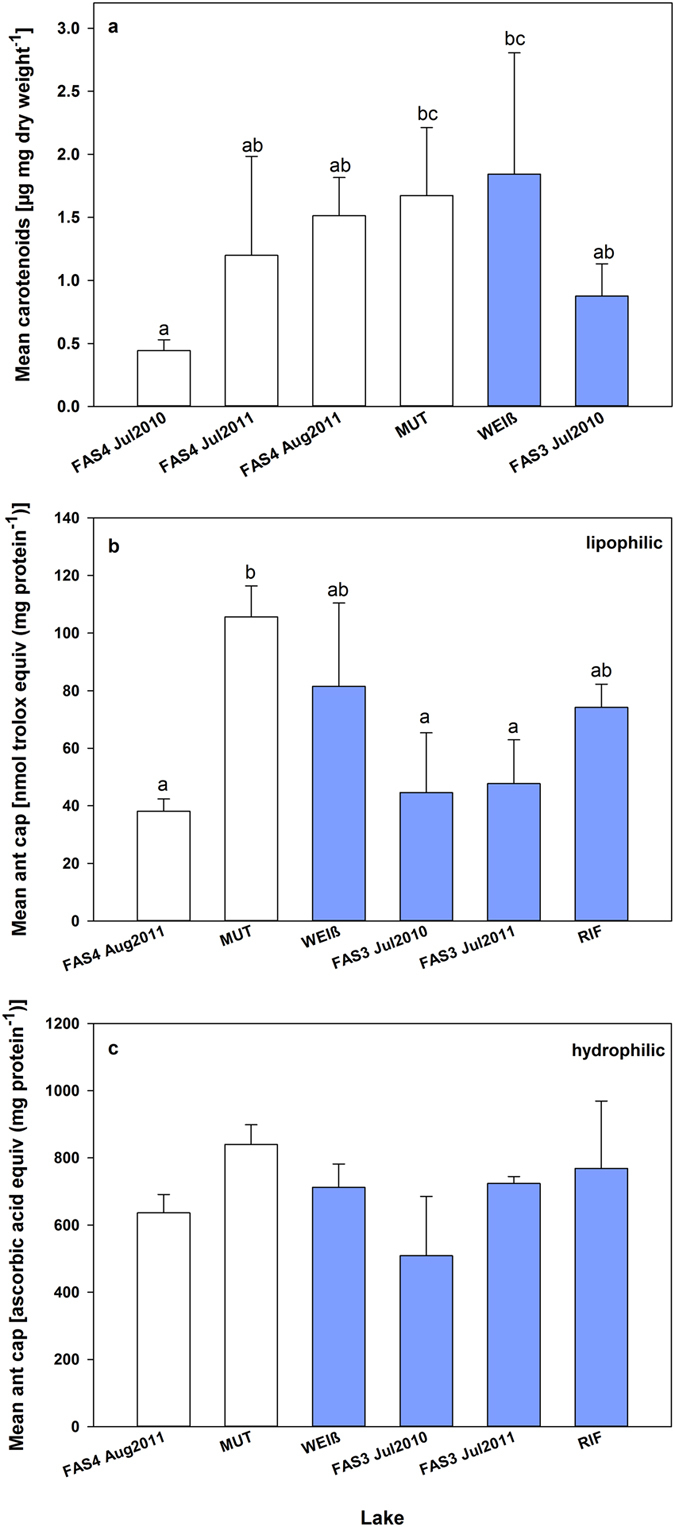
Total mean carotenoid concentrations (µg mg dry weight^−1^) in *Cyclops abyssorum tatricus* copepodid CI to adult life stages (**a**), and mean lipophilic antioxidant capacity (ant cap) (nmol trolox equivalents mg protein^−1^) (**b**), and mean hydrophilic antioxidant capacity (nmol ascorbic acid equivalents mg protein^−1^) (**c**) in *C*. *abyssorum tatricus* copepodid CI–CV life stages from clear (white bars) and turbid glacier-fed (blue bars) alpine lakes sampled during the ice-free period of 2010 and 2011. The lakes are ordered from clear to turbid (abbreviations for the lakes are defined in Table 1). Error bars indicate +1 standard deviation. Different letters above the bars indicate a significant difference found with Kruskal-Wallis one-way ANOVA on Ranks, all pairwise multiple comparison procedures (Dunn’s method, p < 0.05) (**a**), and with one-way ANOVA, all pairwise multiple comparison procedures (Holm-Sidak method, F_5,12_ = 7.31, p = 0.002) (**b**).

Free astaxanthin, esterified astaxanthin, and additionally two unknown carotenoids were detected in the different *C*. *abyssorum tatricus* populations (Table S2). Free and esterified astaxanthin were the dominant compounds, and the maximum proportion of the two unknown carotenoids was ≤7% (Table S2). We were not able to detect any carotenoids in the copepod samples from the most turbid lake (RIF). When comparing the carotenoid levels among lakes, the highest concentrations (pooled life stages CI to adult) were found in the *C*. *abyssorum tatricus* population from Lake WEIß, which were slightly higher than the carotenoid levels of the copepods from Lakes MUT and FAS4 (August 2011) (Fig. 6a). Across the study lakes, *C*. *abyssorum tatricus* carotenoid concentrations were not related to turbidity (r^2^ = 0.01, n = 6, p = 0.851) or UVR transparency (r^2^ = 0.24, n = 6, p = 0.326).

### Antioxidant capacities

The highest levels of antioxidant capacities, both lipophilic and hydrophilic, were measured in the copepod population from Lake MUT (Fig. 6b,c). Similarly, low lipophilic antioxidant capacities were found in clear Lake FAS4 and turbid Lake FAS3, while the antioxidant capacities in copepods from the other glacier-fed lakes were higher (Fig. 6b). Except for lower levels in Lake FAS3 (July 2010), the levels of hydrophilic antioxidant capacities were similar among the different copepod populations (Fig. 6c). No significant relationships were found between the antioxidant capacities of the copepods and optical parameters of the study lakes (lipophilic antioxidant capacities: turbidity r^2^ = 0.02, n = 6, p = 0.800, Z_1%320nm_ r^2^ = 0.01, n = 6, p = 0.899; hydrophilic antioxidant capacities: turbidity r^2^ = 0.08, n = 6, p = 0.596, Z_1%320nm_ r^2^ = 0.003, n = 6, p = 0.919).

## Discussion

When exposed to high levels of UVR, especially in UV transparent and low DOC water bodies such as clear alpine lakes, zooplankton elicit behavioral and physiological responses to reduce the exposure to UVR. In the most UV transparent and fishless lake of our study (Lake FAS4), copepods were absent in water layers down to a depth of 8–10 m (Fig. 1a–c). While copepods are generally considered less responsive to UVR than cladocerans^35^, they show UV avoidance behavior when living in highly transparent lakes, as observed in the copepod *Hesperodiaptomus arcticus* from lakes located in the Canadian Rocky Mountains^10^ and *C*. *abyssorum tatricus* from our Alpine study sites (Fig. 1a–c). In *H*. *arcticus*, the daytime vertical distribution increases with both UVR transparency and depth of food resources (Chl *a*)^10^. Similarly, food resources (i.e., Chl *a* and rotifers) largely explained the variability in weighted mean *C*. *abyssorum tatricus* depths (Fig. 2a,b), while optical parameters (turbidity) became more important when including only the three lakes without fish (Fig. 3). As shown in a previous study^14^, disentangling the effects of UVR and predation on the vertical distribution of copepods is difficult. Though we did not assess the predation pressure, the fish species present in the lakes are known to predate visually and to be planktivorous^36^. However, recent studies from our group on adult *Salvelinus alpinus* and *Salmo trutta* f. *fario* in clear lakes show that they feed mainly on insects (both terrestrial and aquatic) during the ice-free period (Sommaruga R. and Tartarotti B., unpublished data). Nevertheless, the presence of fish (kairomone effects)^37^ may influence the vertical distribution of the copepods. Increased predation pressure is expected in Lake WEIß as it had been recently stocked with fish (Table 1). This might explain the preference of the copepods for the deepest water layers (Fig. 1e), especially since this lake is shallow and the least turbid lake of the glacier-fed study systems (Table 1). We do not have data on the fish biomass present in Lake MUT as this lake has not been stocked with fish for decades; however, low predation pressure is assumed, as the copepods can be found in all water layers, showing no clear depth preference (Fig. 1d). The high turbidity in Lake RIF protects *C*. *abyssorum tatricus* from both UVR and visually-oriented predators, which may explain its uniform distribution in the water column (Fig. 1j). However, the depth distribution pattern found in this lake with very low copepod abundances should be considered with care due to the low sampling volume.

Mineral suspensoids transported by glacial meltwaters are known to affect filter-feeding *Daphnia* by reducing their filtering rate^38, 39^, while other zooplankton taxa such as rotifers feed more selectively, and thus avoid clay and silt particles^38^. Although, to our knowledge, there is no study explicitly focusing on the effect of glacial flour on the feeding behavior of copepods, cyclopoid copepods such as *C*. *abyssorum* are known to be selective feeders^40^. Even if *C*. *abyssorum tatricus* occurs in all of our study lakes, at times of strong turbidity gradients, copepods are found during daytime at depths where turbidity is lowest (Table 2, Fig. 1g) and food levels are high (Table 2). Our results are in line with the findings of Hylander and co-workers (ref. 41) who showed that only in the more transparent part of a deep oligotrophic lake with direct glacier influence, distinct deep chlorophyll maxima and higher copepod abundances are found. Overall, lower zooplankton abundances, albeit higher mean Chl *a* concentrations, in glacier-fed lakes (Fig. 1) suggest that turbidity is an important factor influencing both the number and vertical distribution of copepods in these environments. The co-occurrence of predators and prey in the same water layers offers a refuge from both predators and UVR in clear lakes^7^. In our study, the copepods co-occur with their potential prey (rotifers) (Fig. 2b), at the same time this spatial overlap between predators and prey provides protection from UVR in clear lakes such as Lake FAS4.

**Table 2:** Changes in turbidity, Chlorophyll *a* (Chl *a*), and copepod abundance (*Cyclops abyssorum tatricus* copepodid CI to adult life stages) with depth in Lake FAS3 in July 2010.

Depth (m)	0	1	2	4	6	8	10	12	14
Turbidity (NTU)	4.20	4.45	4.31	4.43	4.27	2.91	2.19	1.00	0.96
Chl *a* (µg L^−1^)	0.45	0.42	0.54	0.78	1.71	4.76	7.57	7.75	7.36
Copepods (Ind L^−1^)	0	0	0	0	0	6.33	6.53	6.67	4.73

Solar UVR is important not only for triggering the diel vertical migration of zooplankton^13^, but also for eliciting photoprotection in phytoplankton and thus in zooplankton. Even though at low concentrations (Fig. 4), sunscreens were also present in the copepods from the most turbid lakes (Lakes FAS3 and RIF; Table S1). In the copepod *Arctodiaptomus jurisowitchi* from a glacier-fed alpine lake in the Himalaya Region with the same 1% attenuation depth (at 320 nm) as found in Lake RIF, MAAs were not detectable^17^. These calanoid copepods, however, seem to rely mainly on pigments as protection against UVR, as very high levels of carotenoids (but not MAAs) were reported for *A*. *jurisowitchi* populations from clear Himalayan lakes^17^. Nevertheless, one might ask why *C*. *abyssorum tatricus* from lakes with high UV attenuation accumulate measurable levels of these UV-absorbing sunscreens. Since copepods are not able to synthesize MAAs *de novo*, MAAs have to be obtained through the diet^42^. Indeed, these compounds were also present in seston samples in all of our study lakes (data not shown). MAA concentrations in phytoplankton are influenced by nutrient availability^43^, especially nitrogen as MAAs are nitrogen-based. Glaciers have been suggested to be sources of nitrogen^44^, thus glacial retreat can lead to additional nitrogen inputs, however, inorganic nitrogen concentrations were similar for the glacier-fed (mean: 157 µg L^−1^) and the clear lakes (mean: 164 µg L^−1^), which differs from observations in lakes of the U.S. Rocky Mountains^44^. Concentrations of phosphorus were even higher in the turbid lakes (1.5 µg L^−1^ mean total dissolved phosphorus concentrations in the glacier-fed versus 0.8 µg L^−1^ in the clear lakes), and even if only a small fraction of the phosphorus is bioavailable^45^, the potential synthesis of MAAs in phytoplankton seems not to be hindered by nutrient limitation in our study systems. A previous study shows that copepod MAA concentrations were related to MAA levels in the phytoplankton of clear alpine lakes when considering a time lag between synthesis and subsequent accumulation of these compounds^33^. We assume that this relationship is similar also for turbid glacier-fed lakes and we speculate that at times of low turbidity, for example soon after ice breakup, phytoplankton synthesize MAAs and are then accumulated by zooplankton. Alternatively, other physiological roles of MAAs than UV protection have to be considered, as these compounds are known to be multifunctional secondary metabolites^46^.

Levels of MAAs were up to ~11 times (~3.5 times on average) lower in the *C*. *abyssorum tatricus* populations from the turbid glacier-fed lakes than from the clear ones (Fig. 4). Copepods from the clearest lake contained the highest mean MAA concentrations, which is in accordance with previous studies reporting high accumulation of these compounds in copepods from clear alpine lakes^23, 33^. Our data reflect the need of UV protection in clear systems and also show high variability among lakes. If this variability reflects MAA availability in the diet, with no further ability to accumulate higher MAA levels in the copepods from turbid lakes with low MAA concentrations in the diet, or if there is no physiological need for higher concentrations in lakes with sufficient photo-protection, still needs to be investigated.

In early nauplii stages (orthonauplii), MAAs are derived from egg-carrying females, as they do not feed, whereas from metanauplii onward copepods accumulate these compounds through their diet. The MAA concentrations in these larval stages are generally more variable as compared to older life stages (ref. 33, this study; Fig. 4) and not related to their food (i.e. Chl *a*-specific levels of MAAs in phytoplankton)^33^.

In a previous study including *C*. *abyssorum tatricus* populations from lakes located across an altitude gradient in the Central Alps, both the altitude and the depth refuge (Z_1%320_/Z_max_) largely (86% each) explained differences in the concentration of MAAs^23^. The lakes of the present study are located at similar elevation, but they differ widely in their turbidity levels (Table 1). The depth refuge explained 67% of the variance in the MAA levels (considering the same life stages as in ref. 22), however, turbidity best explained the variability in MAA concentrations (Fig 5e,f). Light/UVR may be the driver of this non-linear relationship because absorbance due to glacial flour increases exponentially with decreasing wavelength^47^, but not as fast as CDOM^48^.

Interactions involving temperature are complex, influencing not only UVR repair mechanisms (i.e. greater repair rates at higher temperatures), but also metabolic activity or life span^5^. In phytoplankton from a clear alpine lake, the MAA concentrations were strongly related to the lake water temperature^33^, suggesting that the synthesis of MAAs is triggered not only by increased sunlight intensities, but also that temperature affects its rate. However, the same study also showed that solar radiation is more likely to influence MAA contents (both in phyto- and zooplankton) than increased water temperatures alone^33^. Similarly, we found a positive relationship between MAA concentrations of the copepods (copepodid stages CI-CIV, r^2^: 0.49) and the mean water temperature, nonetheless, the correlation was stronger when related to lake transparency (Z_1%320_, r^2^: 0.77; Fig. 5e).

Beside turbidity given by glacial flour, also dissolved organic carbon (DOC) provides protection to planktonic organisms from UVR^49^, probably resulting in less need for photoprotective compounds such as MAAs. Indeed, in Lake MUT, which has an almost two-fold higher mean DOC concentration when compared with the other study lakes (Table 1), MAA concentrations in copepods were ~2.1-times lower than in those of the clearest lake (Fig. 4). Although both lakes are clear systems, the 1% attenuation depth (Z_1%320_) in Lake MUT was only about half that of Lake FAS4 (Table 1), resulting in less influence of UVR also on the behavioral response of the copepods (Fig. 1d). In this lake, the copepods did not avoid the surface waters as they did in highly UV transparent Lake FAS4 (Fig. 1a–c).

The copepods examined in our study showed red pigmentation, except for the copepod population from the most turbid lake (Lake RIF), which was translucent. In general, carotenoid levels were lower than those found in the *C*. *abyssorum tatricus* population from the clear alpine lake Gossenköllesee^50^ or other cyclopoid copepod species from diverse high mountain lakes^34, 51^. This difference may partly be explained by the use of different extraction (DMF with BHT, see Methods) and analytical methods (HPLC), as samples from earlier studies were extracted with ethanol and quantified with a spectrophotometer^15, 50, 51^. Moreover, our carotenoid samples were grouped (CI-adult), which might influence the pigment content, as carotenoid concentrations are known to change with developmental stage. Higher contents (~20–30%) are reported in copepodid and adult life stages than in nauplii^52^. Our data, however, confirm the findings of previous studies, showing that astaxanthin in its free form, as well as its esters are predominantly accumulated in freshwater copepods (refs 15, 17 and 20; this study). A strong correlation between the astaxanthin concentration and the depth refuge is found in copepod populations from Himalayan lakes, with the lowest content in the population from the most turbid system^17^. The carotenoid concentrations of the different *C*. *abyssorum tatricus* populations, however, were not related to turbidity or UVR transparency. Although no carotenoids were detectable in the copepods from the most turbid lake (Lake RIF), relatively high carotenoid concentrations were present in copepods even when coming from turbid ecosystems (Fig. 6a). Support for our results comes from a recent study showing that the carotenoid concentrations were ~2.5 times higher in *C*. *abyssorum tatricus* from turbid FAS3 when compared to the population from clear Gossenköllesee (spectrophotometric scans of ethanol extracts, samples from August 2014; B. Tartarotti unpublished data). The astaxanthin concentration also depends on the copepods’ diet, since the amount of available carotenoids is most likely related to the lakes’ individual phytoplankton community structure. When compared with MAAs, our data indicate that carotenoids have a relatively modest role in direct photoprotection. However, carotenoids provide not only antioxidant defenses (see below), but seem to play other physiological roles. Recently, Schneider and co-workers^53^ showed that the accumulation of these pigments in copepods (*Leptodiaptomus minutus*) from a low-UVR boreal lake is related to the lipid metabolism and reproduction.

The *C*. *abyssorum tatricus* population from Lake MUT, the lake with the highest DOC concentration (Table 1), showed the highest antioxidant capacities of the six study lakes (Fig. 6b,c). Although DOC reduces the direct UV exposure of organisms^49, 54^, it is also a photosensitizer generating ROS in surface waters^55^, which are distributed over the entire water body by mixing processes^27^. Damage by oxidation may therefore be reduced by high antioxidant activities present in the copepods. Hylander and co-workers^56^ suggested that MAAs and carotenoids are complementary photoprotective compounds in these animals, i.e., one is high while the other is low. A similar effect might be seen with MAAs and antioxidants, thus, in the presence of relatively low MAA concentrations, organisms from clear systems may need to enhance their antioxidant capacity. Indeed, the lipophilic antioxidant capacity was significantly higher (~2.8-fold; Fig. 6b) in the copepod population from Lake MUT than FAS4, the lake with the highest MAA levels in the copepods (Fig. 4). Lipophilic antioxidants protect the lipophilic structures within the cells, particularly membranes. Evidently, even the low share of the lipophilic antioxidant capacity (Fig. 6b) as compared to the hydrophilic moiety (Fig. 6c) can cover this need. Due to the very low DOC concentration in the most transparent lake of the present investigation (Lake FAS4; Table 1), external ROS may not be as heavily produced as in environments with higher DOC concentrations such as Lake MUT. Moreover, the high concentrations of MAAs in the copepods from FAS4 may be sufficient to protect the animals from damaging UVR with no further need for high antioxidant capacities. Compared to the bulk antioxidant capacity measured by the total antioxidant capacity, UV protective compounds such as astaxanthin and MAAs may be more reliable indicators of UV related oxidative stress^57^. One hypothesis is that UV responsive antioxidants respond faster to stress caused by ROS than “classical” antioxidants (e.g. ascorbic acid, tocopherols, glutathione, or proline)^30, 58^, and thereby protect the organisms from UV-induced oxidative damage. However, this would need to be tested experimentally.

In glacier-fed alpine lakes, turbidity (mainly given by glacial flour) is an important regulator of UVR attenuation^47^, while DOC and phytoplankton largely influence the UVR transparency of the water column when these lakes turn clear^4, 48^. Our findings show that also the strategies used by the copepods change with varying UVR transparency. When exposed to high fluxes of solar radiation such as in clear lakes, *C*. *abyssorum tatricus* individuals can be found in the deepest strata, and accumulate high levels of UV-absorbing compounds, while in highly turbid lakes they are more evenly distributed within the water column and have low concentrations of MAAs. Even small changes in the water turbidity may influence the vertical distribution and contents of photoprotective compounds, showing that zooplankton respond to fluctuations in their environment, for example, changes caused by the influence of glacial melt water discharge on lake ecosystems.

## Methods

### Sampling sites

We sampled six alpine lakes of different UV transparency located in the Austrian Central Alps once during the ice-free season in 2010 (Table 1). Additionally, two of the six lakes (FAS3 and FAS4) were sampled twice in the following summer. Inorganic turbidity in the lakes WEIß, FAS6, FAS3, and RIF originated from glacial meltwater transporting mineral particles into the lakes, thereby inducing strong UVR attenuation within the water column (Table 1). All study sites are located above the treeline with maximum depths ranging from six to 24 m (Table 1). Three lakes contained fish (Table 1), while invertebrate predators were not observed in any of the lakes.

### Zooplankton sampling and sample processing

For estimating zooplankton abundance and vertical distribution, triplicate samples (5 L per replicate) were taken with a modified 5 L Schindler-Patalas sampler above the deepest part of the lake in 1 to 2 m depth intervals from the surface to close to the bottom of the lake around midday (within 2 h of solar noon; dry weather conditions). The zooplankton were fixed in formalin (4% final concentration). Zooplankton samples for MAAs, carotenoids, and antioxidant capacities were collected with a plankton net (50 µm mesh size) by making vertical net tows around midday. Upon return to the laboratory, copepods for antioxidant capacity measurements were immediately placed into filtered lake water (Whatmann GF/F glass fiber filter) for ~2 hours at 4 °C for gut defecation. Narcotized (CO_2_) copepods (60–100 copepodid CI-CV life stages per sample; triplicates; care was taken to use a similar distribution of the different life stages among lakes) were pooled into microcentrifuge tubes, shock frozen, and stored at −80 °C. As photoprotective compounds such as MAAs are rather stable^42^, live zooplankton for MAA and carotenoid analyses were kept at 4–6 °C and in dark conditions until further processing within 24–48 h. Narcotized copepods were separated into the different life stages (i.e., nauplii, copepodid CI, CII, CIII, CIV, copepodid CV female and male, adult female, egg-carrying female, and male) and transferred into microcentrifuge tubes. The number of individuals per sample ranged from 1 (few cases of CV copepodids or adult copepods) to 30 (nauplii), but generally was ~15 individuals per sample. The samples (triplicates) were immediately frozen at −80 °C.

The different life stages of the copepods as well as the number of rotifers were counted under an inverted microscope (Leitz, Labovert) after sedimentation in Utermöhl chambers. The copepods were identified following the taxonomic key of Einsle (ref. 59). For dry weight estimation, the *C*. *abyssorum tatricus* body length was measured at a magnification of 40× or 100×. Biomass was then calculated according to ref. 60.

### Mycosporine-like amino acids, carotenoids, and antioxidant capacities

MAAs were extracted consistent with the most efficient protocol described for *C*. *abyssorum tatricus* ^61^, with some modifications. Samples were extracted in 400 µL of 25% aqueous methanol (v/v; MeOH) for 2 h in a water bath at 45 °C. At the beginning of the extraction, samples were sonicated (30 s at 40 W) on ice, and stored at −80 °C for following characterization using high performance liquid chromatography (HPLC) the next day. For separation and quantification of MAAs, 80 µL aliquots were injected in a Phenosphere 5 µm RP-8 column (4.6 mm internal diameter ×25 cm, Phenomenex) protected with a RP-8 guard column (Brownlee), for isocratic reverse-phase HPLC analysis. Samples were run with a mobile phase of 0.1% acetic acid in 25% aqueous MeOH (v/v) and a flow rate of 0.75 mL min^−1^. The MAAs in the eluate were detected by online UV spectroscopy. Peak measurements were carried out at 310, 320, 334, and 360 nm in a Dionex system with a diode array detector. Individual peaks were identified by relative retention time, absorption spectra, and by co-chromatography with standards (*Porphyra tenera*). The total content of specific MAAs in each sample was calculated from peak areas, using published molar extinction coefficients (see ref. 23). MAA concentrations were normalized to the dry weight of the copepods, expressed as [µg µg^−1^ dry weight].

Carotenoids were extracted and measured according to a protocol developed by Remias and Lütz^62^, with some modifications. Copepod samples were lyophilized overnight, and then extracted for 3 h in an ice-cooled ultrasonic water bath in 250 or 400 µL cooled dimethylformamide (DMF; HPLC-grade) with 0.1% butylated hydroxytoluene (BHT). Extracts were stored overnight at −50 °C and centrifuged (10 min at 10,000 *g*) before injection in the Agilent 1100 HPLC system. Aliquots (70 µL) were injected in a LiChrospher RP-C18 column (4 mm i.d. ×25 cm) protected with a precolumn. Samples were run with a flow rate of 1.4 mL min^−1^, and a mobile phase of 100% of solvent A (acetonitrile/water/methanol/hexane/tris buffer (0.2 M, pH = 8), 80/7/3/1/1) for 15 min, and a linear gradient to 100% of solvent B (methanol/hexane, 5/1) from 15 to 32 min. The HPLC system was equipped with a diode array detector, the chromatograms were quantified at 440 nm, and individual peaks were identified by co-chromatography with standards (all-*trans*-astaxanthin, canthaxanthin, and β-carotene), by relative retention time, and DAD spectra. Carotenoid concentrations were normalized to the dry weight of the copepods, expressed as [µg mg^−1^ dry weight].

For antioxidant capacity measurements, the copepods were homogenized with glass beads in a Speedmill and centrifuged (12,000 *g*, 4 min) using sodium hydrogen phosphate (0.1 M, pH 6.5) as buffer. The cooled supernatant was directly used to determine the antioxidant capacity of water-soluble antioxidants (e.g., glutathione, vitamin C, phenolic compounds, amino acids, phlorotannins, proteins) or was processed to extract lipid-soluble antioxidants (e.g., β-carotene, tocopherols, polyunsaturated fatty acids, pigments) according to Bligh and Dyer^63^. The antioxidant capacity was analyzed via photo-chemiluminescence in a PhotoChem device (Analytik Jena) based on Popov and Lewin^31^. Antioxidant capacities were related to the protein content of the copepods (measured according to ref. 64), and expressed as nM trolox or ascorbic acid equivalents [mg protein]^−1^ for lipophilic and hydrophilic antioxidants, respectively.

### UV attenuation, turbidity, and Chl *a* measurements

For colored dissolved organic matter (CDOM) absorption measurements, water samples (~0.5 m depth) were collected in precombusted glass bottles and filtered (GF/F; precombusted). Absorbance of filtered lake water was measured with a Hitachi U-2000 double-beam spectrophotometer between 250 and 750 nm at 1-nm intervals using 5-cm or 10-cm fused silica cuvettes (Suprasil I), respectively, referenced against 0.22-µm membrane-filtered Milli-Q water. The absorbance value at 690 nm was used to correct UV absorbance values for the presence of particulate matter. For the clear lakes, absorption coefficients (*a*_*g*_) for the dissolved fraction of lake water were calculated as *a*_*g*_ = 2.303 D/r where D is absorbance and r is the path length in meters. The diffuse vertical attenuation coefficients (*K*_d_) were estimated by the CDOM absorptivity measurements using the model equations described by Laurion and co-workers^4^ for alpine lakes. As solar radiation is scattered by glacial flour, the UVR attenuation would be underestimated when using the CDOM absorptivity to estimate the K_*d*_ for turbid lakes. Thus, for the glacier-fed lakes, we used the equation given by Rose and colleagues^47^ to calculate the K_*d*_ from turbidity values. At Lakes FAS3 and FAS4 (summer 2011), in addition, underwater irradiance-depth profiles were taken with a PUV-501B profiler radiometer. The *K*_d_ in the water column was determined from the slope of the linear regression of the natural logarithm of down welling irradiance (*E*_d_) versus depth (z).

For turbidity and Chl *a* measurements, water samples were collected with a modified Schindler-Patalas sampler from surface to maximum depths (1–2 m depth intervals). Turbidity was measured as nephelometric turbidity units (NTU) using a WTW Turb 430 T calibrated between 0.02, 10, and 1000 NTU. For Chl *a*, water samples of 1.2 to 2.6 L (prefiltered by a 100-µm mesh to remove zooplankton) were filtered through GF/F filters. Chl *a* was extracted in alkaline acetone, measured with a spectrophotometer, and the equation of Lorenzen^65^ was used to calculate the Chl *a* concentrations.

### Data treatment

Daytime weighted mean depths were calculated for *C*. *abyssorum tatricus* (copepodid C1 to adult) using a modified version^66^ of the calculation given by Worthington^67^, WMD = Σ(*d*_*i*_ *n*_*i*_ *l*_*i*_)/Σ(*n*_*i*_ *l*_*i*_) where *d*_*i*_ is the depth sampled, *n*_*i*_ is the number of organisms caught in *d*_*i*_, and *l*_*i*_ is the number of meters represented by the *i* ^*th*^ sample. The *l*_*i*_ values were included because sampling depth intervals were not equal over the whole range. The value of *l*_*i*_ was calculated as the number of meters from half the distance to the sample above (or surface) to half the distance to the sample below (or bottom)^66^. For the samples from the lake surface, a sampling depth of 0.25 m was assumed. Data are reported as mean ± standard deviation, level of significance was set to p < 0.05. Data were not transformed, except for the correlation between turbidity and Z_1%320 nm_ (log transformation of the values). Differences in the copepod mean depths in fishless lakes and lakes with fish were analyzed by a t-test. The significance of differences between MAA concentrations, carotenoid levels, and lipophilic antioxidant capacities of the copepod populations across the lakes was evaluated by one-way Analysis of Variance (ANOVA) with Holm-Sidak posthoc test or Kruskal-Wallis one-way ANOVA on ranks (Dunn’s method) when normality failed. The analyses were done using the Systat software SigmaStat 3.5. For MAAs, the concentrations of early (copepodid CI-CIV) and late life stages (copepodid CV-adult) were grouped together, following feeding preferences in cyclopoid copepods, changing from a dominantly algivorous to a more omnivorous diet. As copepods were rare in some of the lakes, carotenoid samples (copepodid CI to adult life stages) were grouped together to obtain enough data for statistical analyses.

## Electronic supplementary material

Supplementary Information
