# Supplementary material for: Distribution and UV protection strategies of zooplankton in clear and glacier-fed alpine lakes
Source: Sci Rep. 2017 Jul 3;7:4487. doi: 10.1038/s41598-017-04836-w (PMC5495746; doi:10.1038/s41598-017-04836-w)
Supplement: Supplementary file 1 — Supplementary Information [file 41598_2017_4836_MOESM1_ESM.pdf]

Distribution and UV protection strategies of zooplankton in clear and glacier-fed alpine lakes

Barbara Tartarotti, Florian Trattner, Daniel Remias, Nadine Saul, Christian E.W. Steinberg, and Ruben Sommaruga

Table S1. Proportion (%) of the different MAAs in *Cyclops abyssorum tatricus* (averaged over all life stages) of the six study lakes (FAS4: Faselfadsee 4, MUT: Mutterbergersee; WEIß: Weißsee, FAS6: Faselfadsee 6, FAS3: Faselfadsee 3, RIF: Rifflsee). Abbreviations: SH = shinorine, PR = porphyra-334, PI = palythine, AS = asterina-330, US = usujirene, PE = palythene; n.d. = not detectable.

Lake/sampling date	%SH	%PR	%PI	%AS	%US	%PE
FAS4 (2010-07-12)	72.26	n.d.	2.50	14.43	4.70	6.11
FAS4 (2011-07-05)	69.76	0.07	2.72	18.23	1.33	7.89
FAS4 (2011-08-29)	47.54	n.d.	6.66	30.35	1.76	13.70
MUT	39.36	n.d.	15.39	30.66	6.57	8.02
WEIß	80.22	13.30	n.d.	6.48	n.d.	n.d.
FAS6	41.49	58.51	n.d.	n.d.	n.d.	n.d.
FAS3 (2010-07-12)	88.87	3.77	0.23	1.39	0.22	5.52
FAS3 (2011-07-05)	87.38	1.46	0.18	0.66	1.20	9.12
FAS3 (2011-08-29)	82.26	1.13	0.02	1.23	1.25	14.11
RIF	81.24	5.26	0.68	0.35	1.06	11.41

Table S2. Proportion (%) of the different carotenoids in *Cyclops abyssorum tatricus* (averaged over copepodid CI to adult life stages) from the study lakes (abbreviations for the lakes are defined in Table S1). Data are not available for two study lakes (carotenoids were not detectable in Rifflsee (RIF), and there were not enough individuals available from Faselfadsee 6 (FAS6)). Abbreviations: Asta free = free astaxanthin, Asta est = esterified astaxanthin, Car1 = unknown carotenoid #1, Car2 = unknown carotenoid #2; n.d. = not detectable.

Lake/sampling date	%Asta free	%Asta est	%Car1	%Car2
FAS4 (2010-07-12)	100.0	n.d.	n.d.	n.d.
FAS4 (2011-07-05)	43.9	49.4	4.7	2.0
FAS4 (2011-08-29)	43.6	45.1	7.2	4.1
MUT	41.4	49.4	4.6	4.6
WEIß	40.9	44.8	7.1	7.2
FAS3 (2010-07-12)	22.2	68.2	3.7	5.9
